# Artificial intelligence in the intensive care unit

**DOI:** 10.1186/s13054-018-2301-9

**Published:** 2019-01-10

**Authors:** Christopher A. Lovejoy, Varun Buch, Mahiben Maruthappu

**Affiliations:** 1 Cera Care, London, UK; 2 St George’s Hospital, Blackshaw Road, London, SW17 0QT UK

## Introduction

The use of artificial intelligence (AI) in healthcare is receiving increasing interest, driven by a surge in scientific research and funding. AI has shown ophthalmologist-level performance at detecting retinal pathology [1] and can provide individualised treatment decisions for sepsis that could improve patient outcomes [2]. There are many potential applications in the intensive care unit (ICU), particularly given the large amounts of data collected routinely. However, there are some important considerations for ensuring successful implementation.

## Why artificial intelligence?

There is a paucity of positive multi-centre prospective randomised control trials in ICU settings. This reflects the challenge of running studies in such environments, where multiple treatments are given simultaneously to individuals who respond in variable ways based on their individual physiology. The resultant absence of strong guidelines means that clinician decision-making is driven largely by experience and instinct, resulting in significant variability amongst clinicians.

AI could reduce this inter-clinician variability and offer other benefits. AI excels at finding complex relationships in large volumes of data and can simultaneously and rapidly analyse many variables to predict outcomes of interest, such as sepsis or mortality. The modern ICU environment is data-rich, providing fertile soil for the development of more accurate predictive models, better decision support tools, and greater personalisation of care.

## Predictive models and decision support tools

### Severity scoring and mortality prediction

Given the complexity and heterogeneity of ICU patients, scoring systems have been generated to record severity of illness and predict probability of mortality. They can assist in clinical decision-making, comparisons of quality of care, and stratification for clinical trials. However, they do not incorporate variations between departments, regions and countries and perform better on entire ICU populations than on individuals or subsets [3].

AI is well suited for developing algorithms which overcome these limitations and also increase prediction accuracy. The artificial neural network of Dybowski et al. could be re-trained in individual ICUs, tailoring predictions to that unit [4]. Pirracchio et al. [5] and Aczon et al. [6] used multiple machine learning (ML) methods to achieve superior areas under the curve (AUCs) of 0.94 and 0.93 respectively.

### Prediction of sepsis

Early detection and prediction of sepsis enable earlier treatment and better outcomes, yet sepsis is often unclear until late stages. Existing tools have poor predictive accuracy and often rely on time-consuming laboratory results. Desautels et al. found that, in 22,853 ICU stays, systemic inflammatory response syndrome (SIRS), Simplified Acute Physiology Score II (SAPS II) and sequential organ failure assessment (SOFA) had AUCs of 0.609, 0.700 and 0.725 respectively for identifying sepsis at the time of onset [7].

The AI model by Nemati et al. predicted sepsis 12 h before onset with an AUC of 0.83 [8]. Kamaleswaran et al. used multiple ML techniques to identify novel predictive markers [9]. They found that variations in vital signs, processed by AI, could identify children who would develop severe sepsis [9]. Without the wait for laboratory results, earlier treatment is enabled.

### Decision support in mechanical ventilation

Mechanical ventilation is one of the most common interventions in ICU patients. Appropriate levels of sedation and analgesia are important but complicated by significant inter-patient variability. Timing of ventilator removal is also important, as both premature extubation and prolonged ventilation are associated with higher mortality rates. However, a wide discrepancy of practices is seen and accurate prediction is challenging.

An AI tool may enable more personalised sedation and analgesia to reduce inter-clinician variability. The algorithm of Prasad et al. outperformed clinical practice, as measured by regulation of vital signs [10]. Using AI to guide extubation timing is challenging. Such algorithms are trained using outcome data, such as the timing of removal and whether it was successful. However, successful extubation only indicates that it was ready at that point, and doesn't rule out being ready at an earlier stage. This is also true in the reverse for premature removal. Despite this, the algorithm of Parreco et al. predicted the need for prolonged ventilation with an AUC of 0.82 [11]. The AI algorithm of Prasad et al., used to advise when to wean, outperformed clinical practice in terms of number of re-intubations [10].

## Improving data with new technology

Novel types of data, collected by new methods, enable improvement of existing models and development of new tools. Davoudi et al. explored the use of wearable sensors, light and sound sensors, and a camera to collect data on ICU patients and their environments [12]. The authors found that delirious patients were more sensitive to light but not to noise. Such data analysis could improve detection of delirium and enable real-time interventions to improve sleep hygiene [12]. Pickham et al. detected patient positioning with wearables to identify when patients should be turned and thus reduced hospital-acquired pressure injuries [13]. A variety of AI methods allow the processing of these novel types of data, such as convolutional neural networks which excel at analysing visual information.

AI is also taking advantage of the move towards higher-resolution continuous data capture. For example, deep learning analysis of electrocardiogram data, measured continuously in ICU patients, can detect ST-changes [14] and a broad range of arrhythmias [15].

## Important considerations

While AI may enable the development of accurate tools, their introduction must follow careful consideration of real-world clinical utility, efficiency and existing workflows. The use of AI should be appropriately weighted alongside other sources of information and should be validated by well-designed prospective studies before widespread implementation.

Training AI algorithms requires integrated, well-structured data. Many ICUs use a combination of paper and electronic data and do not electronically link together data collected from multiple sources. Data are often incomplete or incorrectly entered. It is also heterogeneous, and many different tests are measured at different times. However, many AI models have achieved accuracy despite these factors.

Healthcare data are sensitive so data security and patient privacy are important considerations. Appropriate consent must also be obtained for data collection, yet many ICU patients lack sufficient capacity until recovery.

## Conclusion

ICU doctors are often required to analyse large volumes of complex, heterogeneous data to make life-critical decisions. AI, if used effectively, could reduce this burden by transforming data into more actionable information. We can use AI to predict adverse outcomes before they happen, better manage highly complex situations, and ultimately allow clinicians to spend less time analysing data and more time harnessing their experience and human touch in delivering care.

## Abbreviations

AI: Artificial intelligence
AUC: Area under the curve
ICU: Intensive care unit
ML: Machine learning
